# Supplementary material for: scDock: streamlining drug discovery targeting cell–cell communication via scRNA-seq analysis and molecular docking
Source: Bioinformatics. 2026 Mar 2;42(3):btag103. doi: 10.1093/bioinformatics/btag103 (PMC12996892; doi:10.1093/bioinformatics/btag103)
Supplement: btag103_Supplementary_Data [file btag103_supplementary_data.docx]

**Supplementary Figure 1. Cell type annotation and distribution of the GSE218563 dataset.**

Each cell type is shown in a distinct color and labeled on the UMAP plot.





**Supplementary Figure 2. Partial visualization of differential signaling in the diabetic nephropathy (DN) group compared with the control group.**

This bubble plot highlights signaling pathways with higher activity probabilities in the DN group. The control group is shown in green, while the DN group is shown in orange.


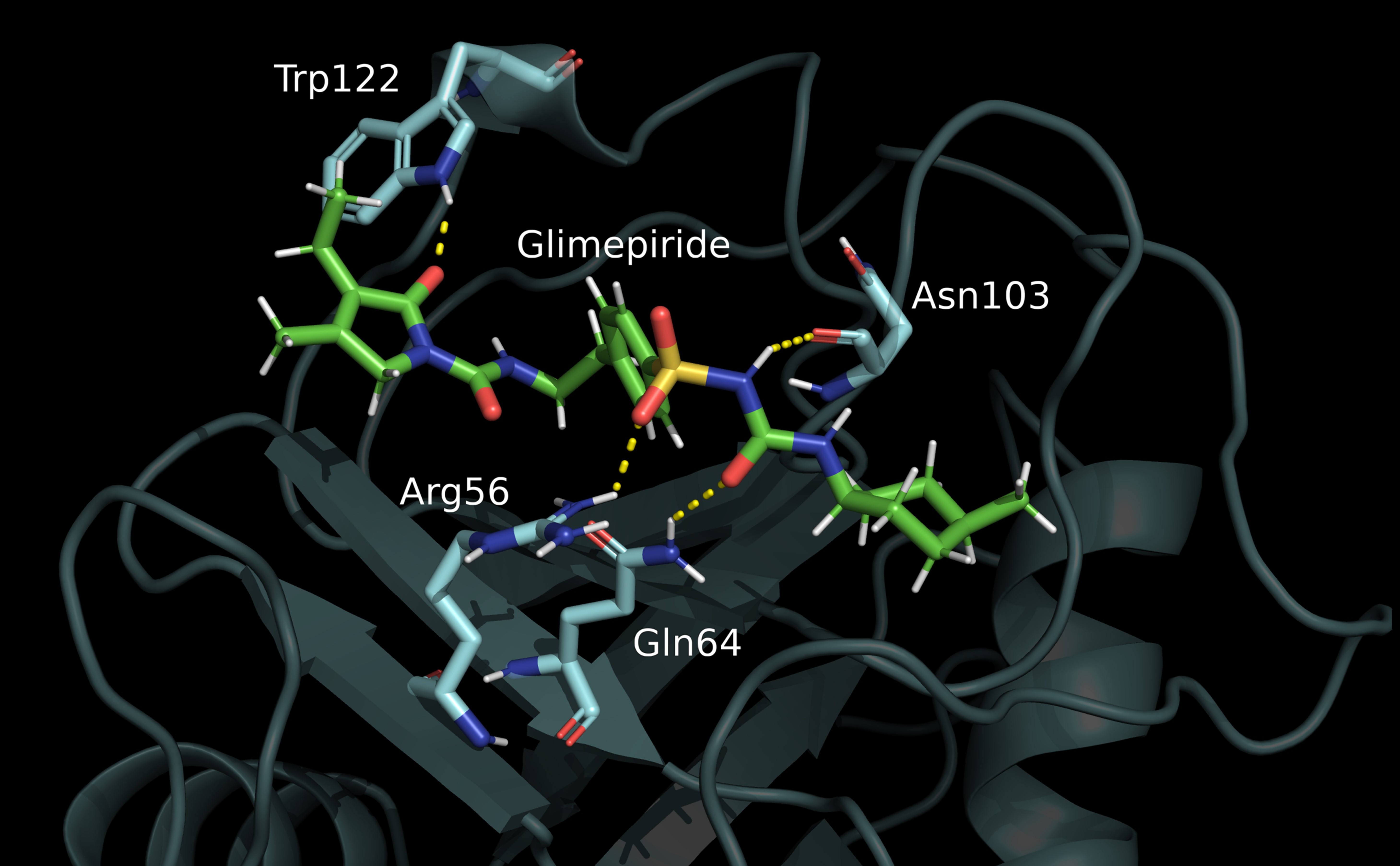


**Supplementary Figure 3. Molecular interaction between glimepiride and the PPIA protein.**

The PPIA protein is shown in cyan, and glimepiride is shown in green. Yellow dashed lines indicate hydrogen bonds between the two molecules.
